# Fertility awareness methods and waiting conduct in idiopathic infertility: a prospective observational study

**DOI:** 10.3389/frph.2026.1753325

**Published:** 2026-03-10

**Authors:** Michele Barbato, Serena Del Zoppo, Fabio Parazzini, Andrea Graziani, Maurizio Guida, Alberto Ferlin, Luigi Frigerio, Giuseppe Grande

**Affiliations:** 1 Sintotermico C.A.Me.N. (Italian National School of the Camen Symptothermal Natural Method for Fertility Knowledge), Milan, Italy; 2 Department of Clinical and Community Sciences, University of Milan, Milan, Italy; 3 Department of Medicine, University of Padova, Padova, Italy; 4 Department of Obstetrics and Gynecology, “Federico II” University Hospital, University of Napoli, Napoli, Italy; 5 Department of Systems Medicine, Unit of Andrology and Reproductive Medicine, University Hospital of Padova, Padova, Italy; 6 Department of Obstetrics & Gynaecology, Hospital Papa Giovanni XXIII, Bergamo, Italy; 7 International Chair in Bioethics “Jerome Lejeune”, Madrid, Spain

**Keywords:** assisted reproductive technologies, fertility awareness methods, idiopathic infertility, infertility, natural family planning methods

## Abstract

**Introduction:**

Couple infertility is a common clinical condition that is too often treated with assisted reproductive techniques (ARTs) without a proper evaluation of both male and female factors. To improve the likelihood of natural conception, fertility awareness methods (FAMs) are widely used.

**Methods:**

We performed a multicenter prospective study enrolling couples with primary idiopathic infertility who were seeking natural conception. Participants were followed for 12 months using FAMs, and their outcomes were compared with those of couples who only used ARTs. The aim of our study was to evaluate the pregnancy rate after 12 months among couples with idiopathic infertility using FAMs compared with those who immediately pursued ARTs. We evaluated 41 couples in the FAM group and 56 couples in the ART group.

**Results:**

In the FAM group, we reported a pregnancy rate (PR) of 51.22%. Among women aged <34 years, we reported a PR of 90.9%, while it decreased to 36.7% among women aged 35–39 years. In the ART group, 10 couples achieved pregnancy (PR 17.8%). Within this group, we reported a PR of 30% among women aged <34 years and 17.4% among women aged 35–39 years.

**Discussion:**

After 12 months of unprotected intercourse without spontaneous conception in women younger than 35 years or after 6 months in women aged 35–39 years, couples should undergo a complete multidisciplinary diagnostic evaluation involving both the male and female partners. If a diagnosis of idiopathic infertility is established at the end of this process, couples (especially younger ones) may be advised to wait an additional 12 months while using FAMs, as no advantage has been observed with direct access to ARTs. They could then be referred to ART if a spontaneous pregnancy is not achieved during this period.

## Introduction

Couple infertility is defined as the inability to conceive after 12 months of regular, unprotected sexual intercourse (1). It is a common clinical condition with negative clinical, economic, and psychosocial impacts, affecting about 20% of couples worldwide (2–5).

In light of the evidence that infertility is a condition affecting the couple as a whole, it is clinically (and even ethically) appropriate to delve into both male factor and female factor infertility (MFI and FFI, respectively) (6–9). In fact, MFI is present, either alone or in combination with FFI, in almost half of the overall cases of couple infertility (4, 9).

Idiopathic or unexplained infertility has been reported in up to 30% of infertile couples (10). Nevertheless, the rate of idiopathic infertility largely depends on the extent of the evaluation prescribed for infertile couples. Indeed, a recent study (7) reported a rate of only 8% of idiopathic couple infertility when both MFI and FFI were thoroughly studied. Therefore, idiopathic couple infertility is a diagnosis of exclusion, which might be proposed following all the possible evaluations of male and female partners. This approach is of paramount importance, as evidence shows that a proper, complete diagnostic work-up often allows identification of the main cause of MFI and FFI. This is particularly true when dealing with MFI, which is often studied solely by semen analysis (6).

MFI might be caused by several risk factors and etiologies (including neoplasms, drugs, infections, inflammatory conditions, varicocele, trauma, hormonal alterations, and genetic abnormalities), therefore requiring further evaluations to properly classify the male partner with a proper diagnosis and identify the leading cause of MFI (4, 8, 9, 11–15). A complete diagnostic work-up of MFI is therefore advisable to guide appropriate therapy for such MFI, for instance, anti-inflammatory, anti-infective, or hormonal therapy, which may improve semen parameters and/or restore natural fertility (4, 6, 7, 9, 16–19).

To improve the probability of conception, fertility awareness methods (FAMs) are widely used, especially in couples with idiopathic infertility. FAMs are based on the knowledge of female physiology and aim to identify fertile and infertile phases of the ovulatory cycle through the observation, recording, and interpretation of various single or combined indicators of fertility, such as cervical mucus characteristics, basal body temperature (BBT), and cervical changes.

Interestingly, in fact, cervical mucus plays a pivotal role in couple fertility. When mucus becomes markedly estrogenic (i.e., characterized by a clear, stretchy appearance and a wet, slippery vulvar sensation), it becomes more penetrable to sperm and increases reproductive potential. Thus, intercourse timed for the days of optimal cervical secretions may increase the fecundity rate (20, 21). Several studies have demonstrated, at a molecular level, a correlation between cervical mucus composition and function, supporting its use as a marker of fertility (22). More specifically, in fertile couples, the probability of conception is maximum from 3 days before to 3 days after the cervical mucus peak day, whereas the best days to be used in infertile couples appear to be from 1 day before to 1 day after the mucus peak day (23). Thus, monitoring cervical mucus may help infertile couples identify the cycle days with the highest probability of conception (24, 25).

These methods are well-tolerated and have been proven useful in many couples (26–28), especially in those without any other clinical problem causing or being associated with infertility, as is the case in idiopathic infertility.

Therefore, the aim of the present study was to evaluate the pregnancy rate in a group of couples with idiopathic infertility after 12 months of FAM use and to compare this result with the pregnancy rate observed in a control group of couples with idiopathic infertility who were immediately referred to assisted reproductive techniques (ARTs).

## Materials and methods

A multicenter prospective study was conducted between 2010 and 2014 and included 41 couples with primary idiopathic infertility who were seeking natural conception. These couples were selected from a population of 128 couples using FAMs for infertility.

The study design was approved by the institutional review board (IRB) of the Ospedali Riuniti di Bergamo, Italy (approval date: 14 September, 2009). The study was conducted in accordance with the guidelines of the Declaration of Helsinki. All participants provided written informed consent.

All couples had been unable to conceive for at least 12 months.

The female partners underwent medical history collection, physical examination, hormonal assessment of ovulatory function, screening for cervical and vaginal infections, and transvaginal sonography, in accordance with current guidelines (10). Evaluation of tubal patency was performed using sonohysterography or hysterosalpingography in women with normal ovarian reserve.

The male partners underwent medical history collection, physical examination, standard semen analysis in accordance with WHO guidelines, and scrotal ultrasonography (1, 4).

According to current guidelines, idiopathic infertility was defined as infertility in couples with normal BMI; apparently normal ovarian function, fallopian tubes, uterus, cervix, and pelvis; age <40 years; adequate coital frequency; and apparently normal testicular function, genitourinary anatomy, and normal ejaculate (10).

Normal ovarian function was defined according to ESHRE guidelines by the presence of normal menstrual cycles, specifically menstrual cycles lasting 24–38 days, menstrual bleeding of up to 8 days, and a variation between the shortest and longest cycles of <7–9 days. Uterine abnormalities were excluded by transvaginal ultrasonography. Hysterosalpingo-contrast sonography (HyCoSy) or hysterosalpingography was used to confirm tubal patency.

Furthermore, according to the guidelines of the European Thyroid Association (29), all patients were tested for thyroid-stimulating hormone (TSH), and only those with TSH levels of 0.30–2.50 mIU/mL were included.

Accordingly, couples were excluded if the female partner was aged >40 years, had a body mass index (BMI) >25 kg/m^2^, had irregular menstrual cycles, had altered thyroid function, had abnormal uterine structure and anatomy on ultrasound (US) evaluation, or had impaired tubal patency.

All male partners underwent semen analysis, scrotal ultrasonography, and hormonal evaluation for follicle-stimulating hormone, luteinizing hormone, total testosterone, sex hormone-binding globulin, and albumin (to calculate free testosterone). Semen microbiological evaluation was prescribed in patients with normal hormonal parameters and normal testicular volumes who presented with signs/symptoms suggestive of semen infection, in patients with normal hormonal parameters and normal testicular volumes who presented with a reduction in sperm parameters (below the 5th percentile), and in patients with alterations in semen volume (below the 5th percentile or above 95th percentile), abnormal semen pH (normal range: 7.2–7.8), altered semen viscosity or fluidification, leukocytospermia, sperm agglutination, presence of antisperm antibodies, or scrotal ultrasound findings suggestive of male genital tract infection or inflammation (4, 7).

We excluded couples from the present study if the male partner exhibited altered semen parameters, reduced testicular volume or abnormal echogenicity, hormonal abnormalities, altered epididymal dimensions or echogenicity, male genital tract infections, or varicocele on scrotal US.

Couples with idiopathic infertility provided written confirmation of their refusal to immediately undergo ARTs and expressed willingness to adopt FAMs by learning the Centro Ambrosiano Metodi Naturali (CAMeN) symptothermal method. Couples were trained by a qualified instructor.

Each instructor had been previously trained over the course of 1 year across 10 Italian S.T. Camen sites for FAMs, involving a total of 17 FAM operators. The recruitment of FAM operators unfolded through several stages. A year and a half before couple enrollment, the project was introduced to FAM instructors via a presentation outlining the scientific rationale for the use of FAM in facilitating conception among infertile couples. They were provided with a dedicated FAM operators' manual containing all necessary materials. Throughout the duration of the project, a check on the enrollment method and the problems encountered during the study phase was conducted.

**Figure 1:**
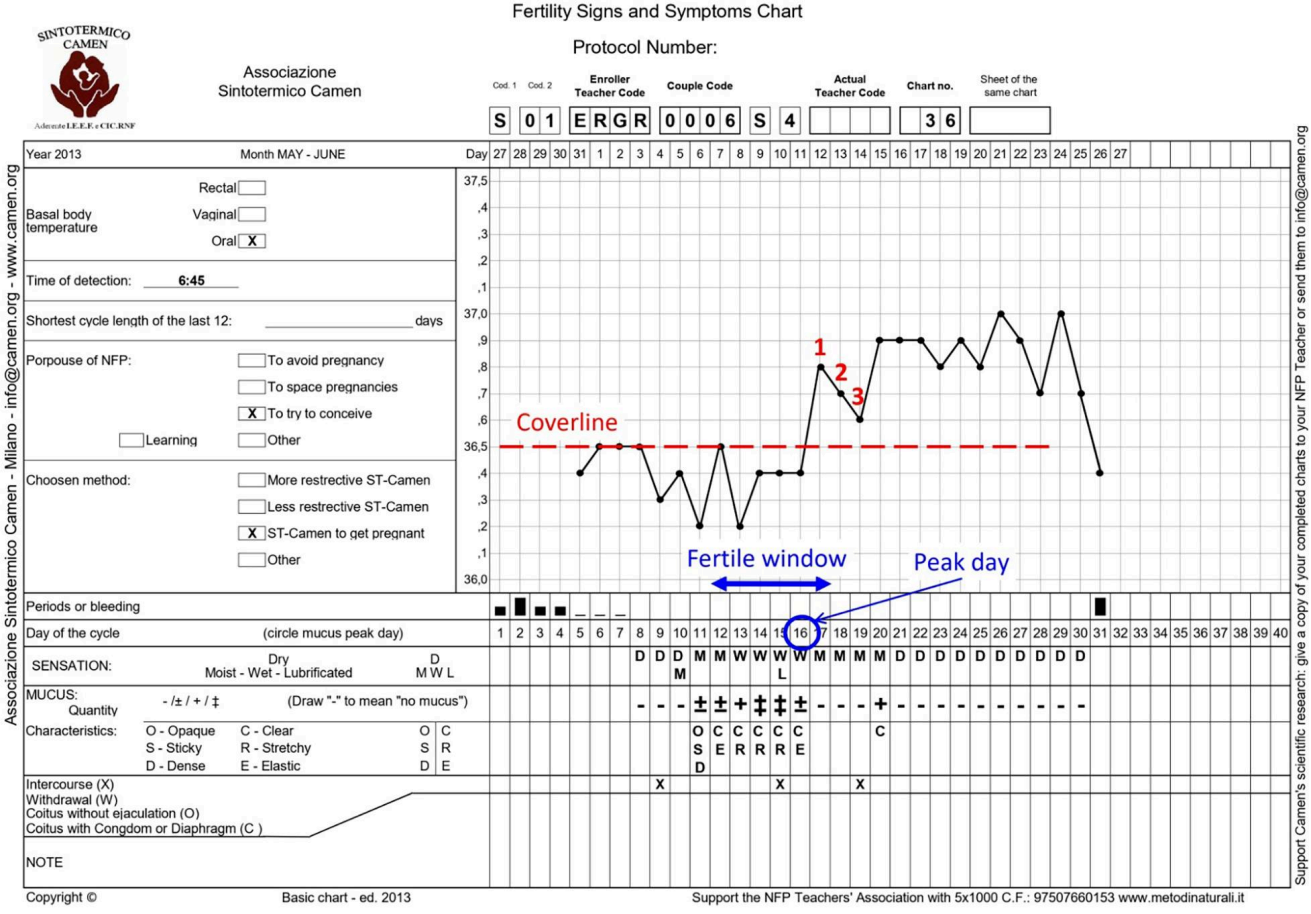
CAMeN symptothermal chart used to register fertility biomarkers in the FAM group.

Participating couples were asked to monitor menstrual cycle biomarkers such as BBT and cervical mucus characteristics and to record acts of intercourse on the chart of the CAMeN symptothermal method (Figure 1).

According to the CAMeN symptothermal method, the fertile window begins on the first day cervical mucus is perceived and/or observed (+/–, +, ++ on the specific chart; Figure 1) and ends on the third day of high BBT after the mucus peak day (30). For the evaluation of temperature, a “cover line” is a line drawn above the highest temperature recorded during the low-temperature phase. Once established, any subsequent temperature value exceeding the cover line by at least one-tenth of a degree is considered high (31).

For couples seeking pregnancy, we used stringent criteria to improve the probability of conception, as follows: (i) the fertile window considers only the most fertile days, defined as the period from the first day cervical mucus became stretchy/elastic, liquid, watery, or reddish, and/or was associated with a wet, slippery, or smooth sensation, until the first day of temperature rise, as identified by the “cover line” (32); (ii) the higher the number of intercourse acts during the fertile window, the higher the probability of pregnancy; (iii) it is desirable to refrain from sex for 4 or 5 days before the prospective start of the fertility window; and (iv) intercourse was preferably advised on days when mucus exhibited its peak fertility characteristics.

In the context of FAM, a couple achieved the status of being “autonomous” once they had acquired the proficiency to apply the method independently. Typically, after two cycles of observation, a couple was deemed autonomous. Subsequently, the study follow-up period commenced with the first menstrual cycle following attainment of “autonomy.” FAM instructors reviewed the charts at 3, 6, 9, and 12 months during follow-up. Consequently, all data were centrally collected.

As is obvious from the above, the project required the involvement of FAM instructors specifically trained in the management of infertile couples according to the project rules (33). Instructors solicited and included the couples, explained the use and rules of the CAMeN symptothermal method, and checked the following aspects: correct understanding and full completion of charts, absence of intercurrent disease (otherwise, referred the couple to a specialist), occurrence of pregnancy and test results, willingness of each couple to continue participation, and the end of the follow-up period (1 year).

As a control group, we enrolled 56 couples with idiopathic infertility, following the same inclusion and exclusion criteria, who underwent ART directly. ART operators followed up with participant couples to check procedure outcomes and record pregnancy and newborn data. The ART-specific details to be collected included the type of procedure, the number of oocytes retrieved, and the number of embryos transferred.

### Statistical analysis

The variables related to the participant couples (e.g., age, previous pregnancies, previous ART cycles, and time spent seeking pregnancy) are presented as frequencies and percentages or means and standard deviations (SDs), as convenient. Differences in characteristics between the two groups were analyzed using chi-square tests or *t*-tests as appropriate.

The cumulative proportions of pregnancies in the FAM group were calculated at 12 months. The cumulative proportions of pregnancies in the ART group were calculated over a complete ART cycle, defined as all transfer attempts of fresh and frozen-thawed embryos resulting from one episode of ovarian stimulation (34).

All statistical tests were two-sided, and differences were considered statistically significant at *P* < 0.05.

## Results

Table 1 reports the characteristics of the studied groups. Couples in the ART group had a longer history of infertility and a higher prevalence of previous failures in ART cycles.

**Table 1:** Characteristics of the studied populations.

Clinical characteristics	FAM group (*n* = 41)	ART group (*n* = 56)
Age (female) (years; mean ± SD)	33.80 ± 4.02	34.18 ± 3.71
Age (male) (years; mean ± SD)	37.07 ± 5.04	38.54 ± 5.15
Time of research (months; mean ± SD)	23.20 ± 20.28	36.03 ± 18.55*
Previous ART cycles (*n*; %)	2; 4.88%	23; 34.32%*

ART, assisted reproductive technique; FAM, fertility awareness method; SD, standard deviation.

* *p* < 0.05.

In the FAM group, we reported 21 spontaneous pregnancies, with a pregnancy rate of 51.22%. In detail, among patients aged <34 years (*n* = 11), we reported 10 spontaneous pregnancies [pregnancy rate (PR) 90.9%]. For patients aged 35–39 years (*n* = 30), the pregnancy rate was reduced to 36.7% (*n* = 11).

Pregnancies in the FAM group were obtained after 5.9 ± 3.9 months of observation.

**Figure 2:**
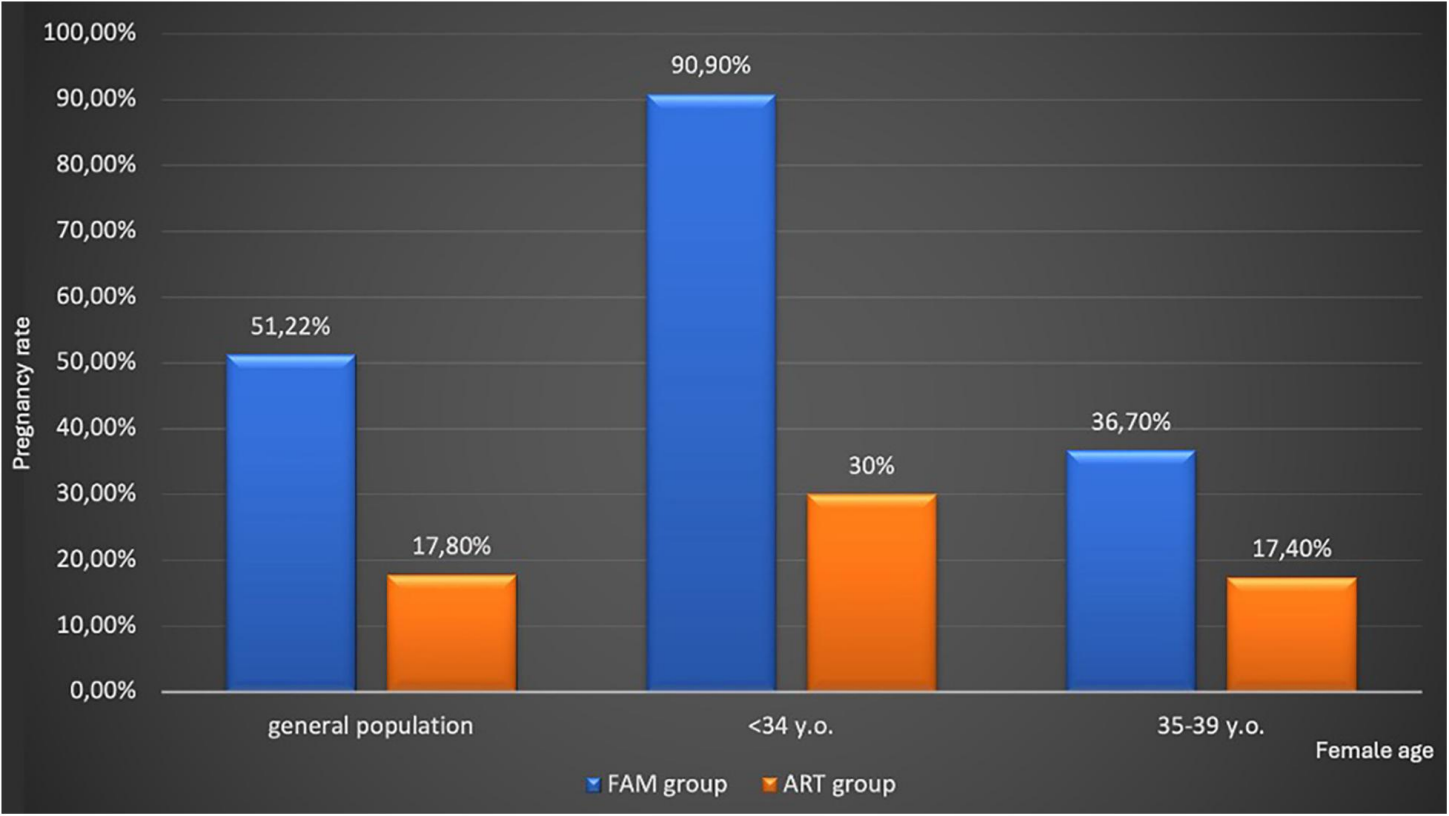
Pregnancy rate in the general population and after stratification for female age. ART, assisted reproductive technique; FAM, fertility awareness method. All data were statistically significant between the FAM and ART groups (*p* < 0.05).

Among the 56 couples in the ART group, *in vitro* fertilization (IVF) was performed in 40 couples and intracytoplasmic sperm injection was performed in 16 couples. Supplementary Table S1 reports data about ART cycles in these couples. Ten couples reported a pregnancy in the ART group (pregnancy rate 17.8%). Among women aged <34 years (*n* = 10), we reported three pregnancies (PR 30%), while in the group of 36 women aged 35–39 years, we reported eight pregnancies (PR 17.4%). Figure 2 summarizes the pregnancy rate in the general population and after stratification by female age.

Supplementary Table S2 reports the clinical criteria of the group of couples who, according to our results, are not yet candidates for medically assisted reproduction and for whom a waiting period combined with FAMs can be recommended.

## Discussion

Concerns about the overuse of ARTs have arisen over the last 20 years due to several factors, including possible short- and long-term safety, economic considerations, and a lack of evidence about effectiveness in specific populations (such as idiopathic infertility) (35–39). In addition, ARTs might pose ethical concerns for certain couples, which should be respected and considered to build and maintain a healthy connection with patients and to support informed, mature decision-making. Previous data have reported the importance of a complete diagnostic and therapeutic flowchart, including the study of the male infertility factor, to restore, when possible, natural fertility (7).

ARTs should therefore be considered within a wider perspective of infertility treatment, considering the clinical situation, the couples’ desires and their ethical choices, and only after a sufficient number of attempts and etiological therapies; this approach is also consistent with ethical principles of distributive justice, fairness, and prioritization.

From this perspective, whether patients with unexplained infertility can benefit from immediate ART treatment or could afford a waiting time in the hope of achieving spontaneous conception represents an open dilemma (40). Previous studies seem, in fact, to suggest that in couples with unexplained subfertility, IVF increases the 1-year chance of conception compared to expectant management, especially for women aged <34 years (41). In contrast to these data, our study suggests that, especially when the woman is younger than 34 years, a spontaneous conception may be achieved in a great proportion of couples after an additional year of intercourses supported by fertility awareness methods. As demonstrated, in women <40 years, and especially in those younger than <34 years, the advantage of ARTs over an additional year of waiting time period combined with FAM cannot be defined in terms of pregnancy rate.

Previous studies reported the efficacy of FAM methods in populations of infertile couples, demonstrating that FAMs were associated with a pregnancy rate of up to 38% after 8 months of use (42). However, it is to be underlined that these studies have several biases since couples had different identified causes and/or conditions associated with infertility. Furthermore, FAMs are often associated with treatment strategies aimed at the restoration of natural fertility both in male and female partners (7, 43, 44); therefore, it has been widely demonstrated that, in infertile couples, the association of FAM methods with a complete diagnostic and therapeutic (medical and surgical) approach aimed at the restoration of natural fertility is associated with a pregnancy rate of about 40% after almost 1 year of follow-up. However, this is the first study to use FAMs in couples with idiopathic infertility, thus excluding any possible bias due to the presence of other causes/factors of infertility and demonstrating the real efficacy of FAM, regardless of any possible confounding factors. Furthermore, future studies will be necessary to compare pregnancy rate in infertile couples followed for infertility with a multidisciplinary path of care including gynecological and andrological evaluation and treatment, associated or not with FAM, thus demonstrating the impact of FAM within an integrated model of care for the restoration of natural fertility in infertile couples.

Our data, moreover, puts into question the clinical significance of idiopathic infertility. In fact, previous data demonstrated that idiopathic infertility is a rare condition, affecting about 8% of infertile couples, if a complete diagnostic workflow is performed in both female and male partners (7). Here, we demonstrated that, particularly among couples in which the woman was younger than <34 years, 90% of the couples with true idiopathic infertility might conceive spontaneously after an additional year of unprotected intercourse combined with fertility awareness knowledge. We therefore proposed a clinical classification of infertile couples in Supplementary Table S2 to identify a population of couples who may benefit from a waiting approach supported by FAMs. Therefore, only a few couples with true idiopathic infertility at the end of a complete diagnostic evaluation of both partners, and after an additional year of waiting time with FAMs, might need access to ARTs, resulting in cost savings for couples and/or for the health service and reduced invasiveness for patients. This evidence is consistent with previous case series, in which an increase in waiting time (up to 18 months) led to more spontaneous conceptions and reduced the adverse effects of prolonged waiting on the take-up rate for treatment and on the chance of success if ARTs are performed (45).

Although the study has limitations, including a small sample size and the absence of a waiting group without FAM learning, our data suggest a specific role for FAM in couples with idiopathic infertility in the waiting group.

## Conclusion

In conclusion, following 12 months of regular, unprotected intercourse without spontaneous conception in women younger than 35 years or after 6 months in women aged 35–39 years, couples should undergo a complete multidisciplinary diagnostic workflow involving both the male and the female partner (a complete and simultaneous diagnostic work-up of both FFI and MFI). If at the end of the process a diagnosis of idiopathic infertility is given, couples (especially the younger ones, being defined by the presence of a woman aged 35 or less) might be addressed to a period of 12 months of waiting time, associated with FAM, since no advantage has been observed by direct ART access. Such couples might eventually be referred to ARTs, according to couples’ desires and their ethical perspectives, only if a spontaneous pregnancy is not achieved following this additional period.

## Data Availability

The original contributions presented in the study are included in the article/Supplementary Material, further inquiries can be directed to the corresponding author.
