# Supplementary material for: Fertility awareness methods and waiting conduct in idiopathic infertility: a prospective observational study
Source: Front Reprod Health. 2026 Mar 10;8:1753325. doi: 10.3389/frph.2026.1753325 (PMC13008926; doi:10.3389/frph.2026.1753325)
Supplement: Supplementary file 1 [file Table1.docx]

**Supplementary Table 1.** Data about ART cycles performed

| N. of oocytes picked-up per patient (mean ± SD) | 3.4 ± 2.0 |
| --- | --- |
| N. of fertilized oocytes per patient (mean ± SD) | 2.2 ± 1.4 |
| Transferred embryos | 1.7 ± 1.1 |

**Supplementary Table 2.** Criteria to identify couples with possible spontaneous conception after waiting time and FAM

| **Female partner** (10,29) | |
| --- | --- |
| Age | <34 y.o. |
| BMI | 18.5 - 24.9 |
| Menstrual cycles | regular menstrual cycle to be 24–38 days, up to 8 days in duration, and shortest to longest cycle variation of <7–9 days |
| Ultrasound | Excluding uterine anomalies |
| Hysterosalpingo-contrast-sonography (HyCoSy) or hysterosalpingography (HSG) | Tubal patency |
| TSH | 0.3 - 2.5 mUI/L |
| **Male partner** (4) | |
| Total sperm count | > 39 x 10^6^ |
| Progressive sperm motility | >32% |
| Total motility | >40% |
| Sperm morphology (normal forms) | >4% |
| Vitality | >58% |
| FSH | 1.5-8 UI/L |
| LH | 1-9.4 UI/L |
| Testosterone | 12-36 nmol/L |
| Calculated free testosterone | >0.220 nmol/L |
| Scrotal ultrasound | Normal testicular volumes (right >12 ml and left >11 mL), normal echogenicity, normal epidydimis dimensions and structure, no varicocele |
| Semen microbiological evaluation (if suspected) | negative |
